# Acceptability Analysis of 3D-Printed Food in the Area of the Czech Republic Based on Survey

**DOI:** 10.3390/foods11203154

**Published:** 2022-10-11

**Authors:** Karolina Tesikova, Lucie Jurkova, Simona Dordevic, Hana Buchtova, Bohuslava Tremlova, Dani Dordevic

**Affiliations:** 1 Department of Plant Origin Food Sciences, Faculty of Veterinary Hygiene and Ecology, University of Veterinary Sciences, Palackeho Trida 1946/1, 612 42 Brno, Czech Republic; 2 Department of Animal Origin Food and Gastronomic Sciences, Faculty of Veterinary Hygiene and Ecology, University of Veterinary Sciences, Palackeho Trida 1946/1, 612 42 Brno, Czech Republic

**Keywords:** 3D food printing, questionnaire survey, consumers, additive manufacturing, novel food, attitudes

## Abstract

The aim of the research was to observe consumer perceptions of 3D food printing and to highlight possible applications of this production. The questionnaire survey took place in the Czech Republic and was attended by 1156 respondents. The questionnaire was divided into six sections: (1) Socio-Demographic Data; (2) 3D Common Printing Awareness; (3) 3D Food Printing Awareness; (4) 3D Food Printing, Worries and Understanding; (5) Application; (6) Investments. Although awareness of 3D food printing is increasing, a very small fraction of respondents had encountered printed food in person (1.5%; *n* = 17). Respondents expressed concerns about the health benefits and the reduced prices of novel foods, and they perceived printed foods as ultra-processed foods (56.0%; *n* = 647). Concerns have also been raised about job losses due to the introduction of new technology. On the contrary, they perceived that quality raw materials would be used to prepare printed foods (52.4%; *n* = 606). Most respondents believed that printed foods would be visually appealing and would find application in several food industry sectors. Most respondents believed that 3D food printing is the future of the food sector (83.8%; *n* = 969). The gained results can be helpful for 3D food printer producers, as well as for future experiments dealing with 3D food printing issues.

## 1. Introduction

The 3D printing technology is a controlled robotic process in which a 3D object is created in layers according to a template from a computer CAD program or from downloaded 3D platforms (e.g., Ponoko, Sculpteo, Shapeways, Thingiverse) [1]. Three-dimensional printing creates objects without any geometric constraints and that cannot be produced by other production processes. Three-dimensional printing is becoming more and more popular because its price keeps decreasing and new applications are constantly being found. This process is also known as Fused Deposition Modeling (FDM) [2,3].

The primary use of 3D food printing is in the custom culinary creation of decorations and food stuffs with unique shapes. The potential of 3D food printing exceeds the boundaries of creating objects of non-traditional forms. With this technology, it is possible to produce food adapted to the requirements of the individual, i.e., lifestyle, health status, and taste preferences [4,5]. Three-dimensional food printing can be applied to a variety of food materials such as chocolate, cheese, dough, hydrogels, etc., [6,7]. Three-dimensional food printing offers a number of benefits. An example is the reduction of food waste by only printing the required amount and processing food that is usually discarded (e.g., imperfect fruits and vegetables) [8].

There is some evidence that consumers are cautious about consuming foods produced by means of new food technologies. The refusal and avoidance of food produced using a new technology is called food neophobia [9,10,11,12,13]. Before launching a new food technology or product, a thorough investigation into consumer opinion and approach is recommended [14].

Although a number of new food technologies have been developed that deal with the 3D printing of matrices made of food material, very little research has been done on how people perceive 3D-printed food [14]. The first attempt to understand 3D food printing is a study by Lupton and Turner [15]. The results of this study show that consumers lack knowledge about 3D printing as well as 3D food printing. Several research participants were concerned about the consumption of 3D-printed foods because they believed that 3D-printed foods were very unnatural or dangerous to eat. Even after communicating the benefits and impacts of 3D food printing in various sectors, the skepticism of most participants was not overcome. In 2020, Manstan and McSweeney [16] conducted a survey on the acceptance of 3D food printing. The results of this study show that half of the respondents say that food produced by 3D printing is acceptable and that 3D food printing can reduce the cost of food production. However, respondents who disagreed with this statement and were not willing to consume 3D-printed foods, claim that 3D-printed foods have more health benefits and are less processed than foods from conventional production (baked/cooked foods). The study conducted by Caulier et al. [17] suggested that the repeated consumption of 3D-printed food increased its acceptability to consumers. 

The aim of the research was to overview consumers’ acceptance of 3D food printing among Czech consumers and how their demographic characteristics influence respondents’ perception toward 3D food printing.

## 2. Materials and Methods

The questionnaires were carried out both in-person (mainly at the University of Veterinary Sciences in Brno, Czech Republic) and online (written in the Czech language with the usage of Google forms/docs) (the questionnaire is included in the Supplementary Materials). The final number of respondents in the sample was *n* = 1156. Data collection was performed from September 2020 to March 2021. The questionnaire was divided into the following sections: Socio-Demographic Data; 3D Common Printing Awareness; 3D Food Printing Awareness; 3D Food Printing, Worries and Understanding; 3D Food Printing, Application; 3D Food Printing, Investments. Socio-demographic data included issues related to sex, age, education, social status, income group, residence, special diets, and places of usual eating. Data from the socio-demographic section are clearly presented in Table 1.

### Statistical Analysis

IBM SPSS 20 software (IBM Corporation, Armonk, New York, NY, USA) was used to conduct the statistical analysis. Data comparison was performed based on the Chi-square test, measuring the agreement between actual counts and expected counts and assuming the null hypothesis.

## 3. Results and Discussion

A total of 1156 respondents took part in the questionnaire survey. The majority of respondents were students, women, and people with a secondary education (Table 1).

It follows that the lower age groups have the highest representation. Therefore, the distribution of respondents is not completely uniform and can be taken into the considerations that further questionnaire surveys will be needed, focusing on the elderly population.

### 3.1. Three-Dimensional Common Printing Awareness

**Figure 1:**
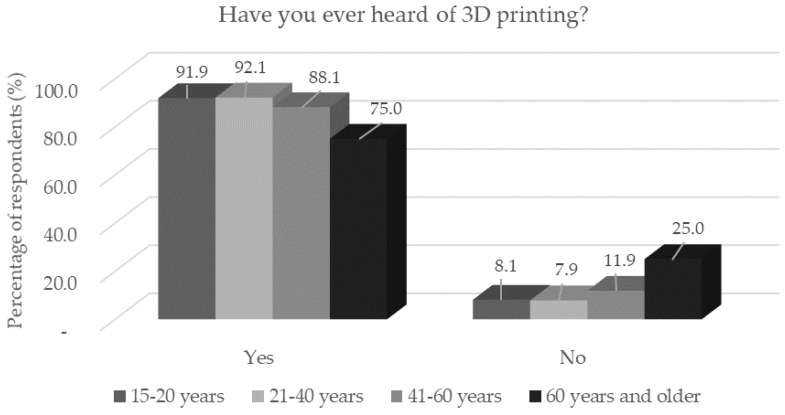
Respondents’ awareness of 3D printing (*n* = 1156) by age category in response to the question: "Have you ever heard of 3D printing?”.

In the conducted survey, most respondents stated that they had heard of 3D printing. Respondents are able to gain high awareness of 3D printing through the widespread use of social networks [18,19]. Compared with the study by Mantihal et al. [18], where 2/3 of the respondents had heard about 3D printing, we can assume that awareness of 3D printing is growing, although the study was conducted in Australia. Although awareness of this technology was high, the results showed a decrease in awareness with increasing age (*p* < 0.05) (Figure 1). Better information was also confirmed among male respondents, single respondents, and respondents with a primary and a university education degree. Statistically significant (*p* < 0.05) differences between these groups were found. It can be assumed that the group of respondents with an elementary education is dominated by young people and students who may be more active on social networks [20], so their awareness of 3D printing may thereby be increased.

Despite the high awareness of 3D printing, active link searching, and the professional literature, 20% of respondents had dealt with it. Statistical significance (*p* < 0.05) was manifested, especially among men, who are more involved in 3D printing than women; although, this result may be influenced by the uneven distribution of respondents where the majority of respondents were women. The highest percentage of respondents interested in 3D printing was monitored in the category of respondents with a basic education, where statistical significance was not confirmed. The percentage of respondents to each question is shown in Table 2.

### 3.2. Three-Dimensional Food Printing Awareness

Despite previous questions, where better awareness of 3D printing has been demonstrated, awareness of 3D food printing has fallen sharply. These results are consistent with the results of the study by Brunner et al. [14], which state that consumers have a relatively low prior knowledge of 3D food printing.

**Figure 2:**
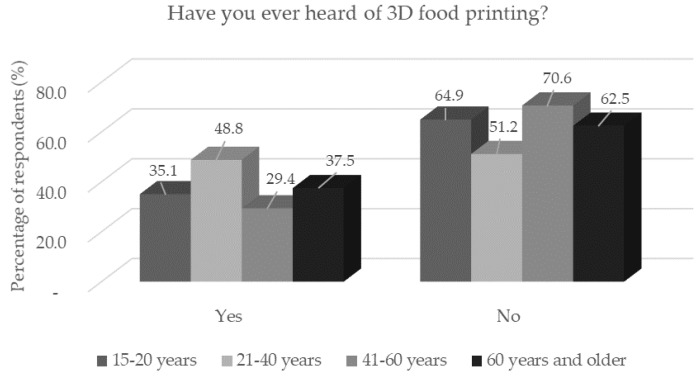
Respondents’ awareness of 3D food printing (*n* = 1156) by age category in response to the question: “Have you ever heard of 3D food printing?”.

**Figure 3:**
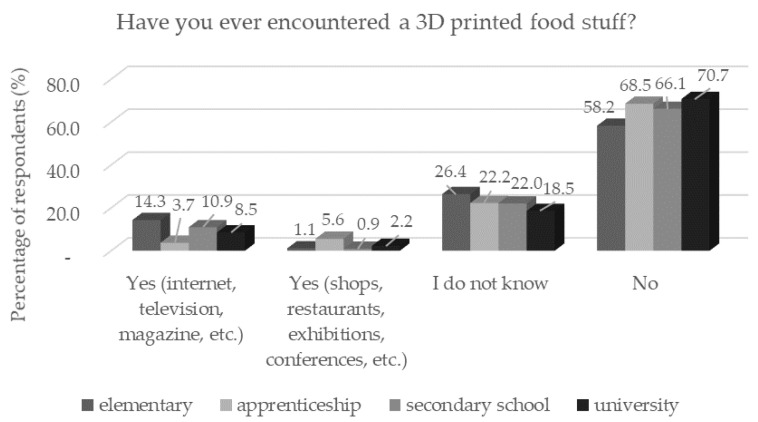
Respondents’ awareness of 3D food printing (*n* = 1156) by education in response to the question: “Have you ever encountered 3D printed food stuff?”.

Lower awareness was also statistically significantly (*p* < 0.05), confirmed in the lowest and the highest age categories (Figure 2). In contrast, statistically significantly (*p* < 0.05) more men than women had heard about 3D food printing, which was expected. A very small fraction of respondents (1.5%) encountered 3D-printed food in person. Through the media, more respondents (*p* < 0.05) with basic education had encountered 3D-printed food. This answer corresponded to the prediction that respondents with a basic (Figure 3) education were composed of adolescents who may be more active on social networks. To raise awareness of 3D food printing, producers of 3D-printed foods should focus on media promotion before launching them. The percentage of respondents to each question is shown in Table 2.

### 3.3. Three-Dimensional Food Printing, Worries and Understanding

**Figure 4:**
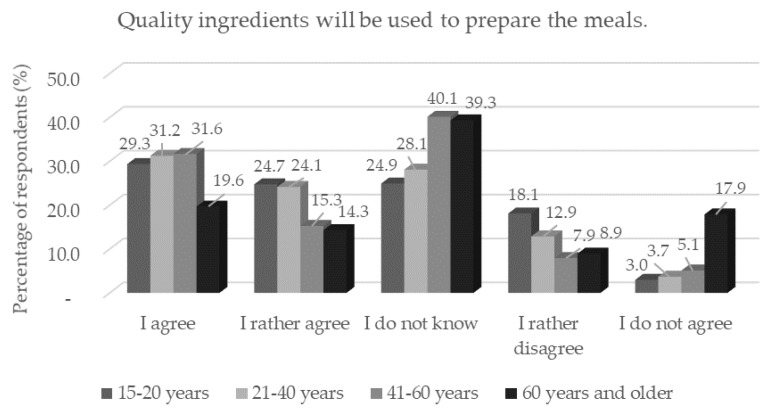
Respondents’ worries and understandings (*n* = 1156) by age category in response to the statement: “Quality ingredients will be used to prepare the meals.”.

Most respondents believe that high-quality raw materials of plant or animal origin will be used to prepare 3D-printed meals. Respondents under the age of 40 (*p* < 0.05) (Figure 4) (*n* = 504) and respondents who had single status (*p* < 0.05) (*n* = 500) were particularly in favor of this statement. Here, it can be concluded that, even though it is a new technology, a large number of respondents did not perceive 3D food printing as a technology that would affect the production of poorer quality food; especially the younger age category confirmed this. One third of respondents had a negative reaction to the health safety of 3D-printed food, and a certain number of respondents were unsure (Table 3).

**Figure 5:**
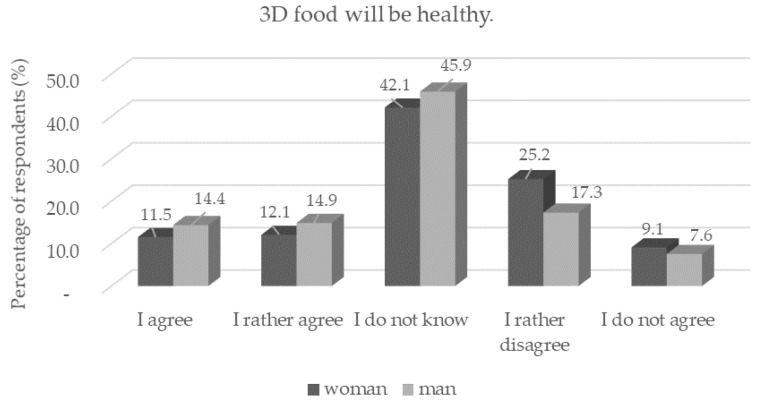
Respondents’ worries and understandings (*n* = 1156) by gender in response to the statement: “3D food will be healthy.”.

This decline in confidence is consistent with a study by Lupton and Turner [15,21], who studied perceptions of the health of raw and printed carrots. Many respondents in this study perceived printed carrots as less healthy, although printed carrots were made from carrot puree and shaped using a 3D printer. Therefore, it can be concluded that any intervention in the food can arouse distrust in the respondents. Women (*n* = 176) were statistically significantly (*p* < 0.05) more distrustful of the health benefits of 3D foods than men (Figure 5).

According to the current information, 3D-printed food is based on starch, sugar, butter, and gelling agents, which are processed into a printing matrix in a powdered form [22]. Therefore, 3D-printed food can be considered as an ultra-processed food. Some studies indicate that ultra-processed food is a symbol of unhealthy food [23,24]. Ultra-processed food is associated with unhealthy products high in fat, sugar, salt, and high-energy value [25]. More than half of the respondents believed that 3D-printed foods would be industrially processed foods, especially respondents aged 41–60 (*p* < 0.05). Several studies suggest that the nutritional value of food plays an important role in choice for the elderly population [26] and for women [27]. Labeling 3D-printed foods as ultra-processed foods could reduce confidence in the above-mentioned populations due to less awareness of the technology.

**Figure 6:**
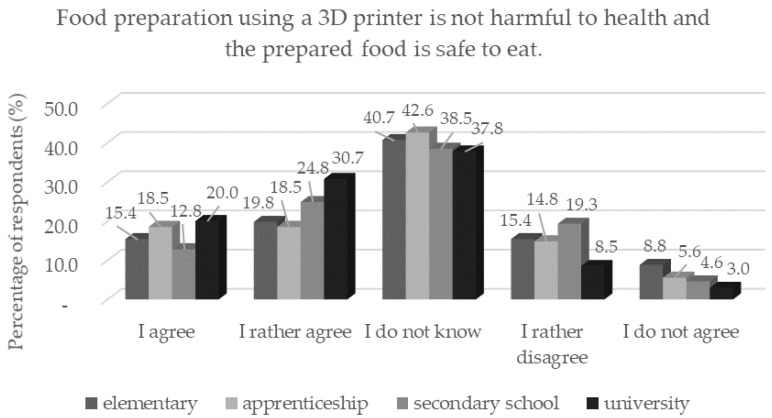
Respondents’ worries and understandings (*n* = 1156) by education in response to the statement: “Food preparation using a 3D printer is not harmful to health and the prepared food is safe to eat.”.

A higher distrust toward the 3D technique among women was statistically significant (*p* < 0.05); also demonstrated in the question of health safety and safe consumption of printed foods. In contrast, respondents with a university degree (*n* = 137) statistically significantly (*p* < 0.05) trusted printed foods more (Figure 6). It has been shown that consumers who trust the food industry and science, find it easier to accept foods processed with new technologies [5]. Therefore, trust in professionals is an important factor in the acceptance of new innovative products by the general public [28]. The results of the study by Evans et al. [29] show that consumers rate foods that have undergone a physical change as more natural than foods exposed to chemical processes.

**Figure 7:**
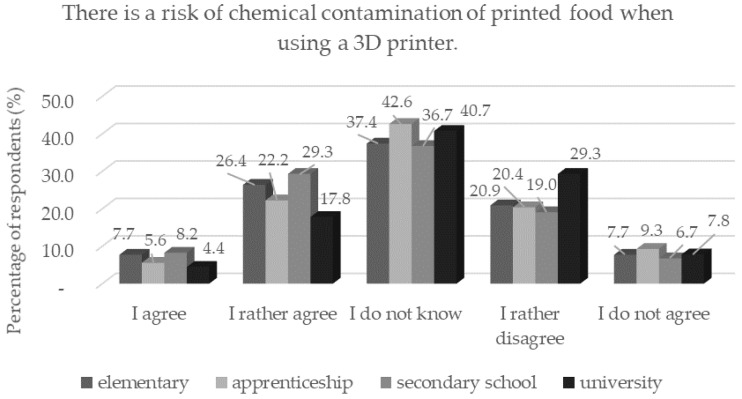
Respondents’ worries and understandings (*n* = 1156) by education in response to the statement: “There is a risk of chemical contamination of printed food when using a 3D printer.”.

The risk of contamination is closely linked to food safety. During food printing, the matrix comes into contact with parts of the 3D printer and contamination may occur if hygiene requirements are not met or if the wrong printer material is selected [30]. Approximately one third of respondents are concerned about microbial and chemical contamination during the preparation of printed meals. Respondents with a secondary education (*n* = 278) had the greatest concerns about chemical contamination of printed foods (*p* < 0.05), while respondents with a university degree (*n* = 100) considered 3D printing to be safe in terms of chemical contamination (Figure 7). Already in the previous results of this study, it was shown that respondents with a university degree trust more in printed foods.

As consumers prefer natural food and food containing as few additives as possible [29], it is necessary to distinguish between printed meals for the general public and meals intended for therapeutic intervention [31].

**Figure 8:**
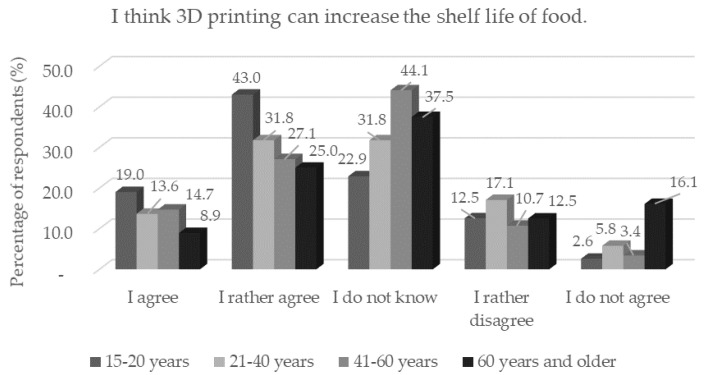
Respondents’ worries and understandings (*n* = 1156) by age category in response to the statement: “I think 3D printing can increase the shelf life of food.”.

One third of the respondents believed that more additives would be used in 3D food printing than in traditionally produced foods. Respondents with a basic education and respondents with single status believed this statement significantly (*p* < 0.05). These respondents also showed a greater awareness of 3D printing technology. Respondents with single status agreed more (*p* < 0.05) that printed foods would be more durable. The same trend (*n* = 336) was observed in respondents in the age category of 15–20 years (*p* < 0.05) (Figure 8).

More than half of respondents did not believe, or were not sure, that 3D food printing could reduce supply costs and thus reduce the price of printed food. At the same time, it was observed that only a third of respondents believed that 3D food printing could have a positive impact on the environment. There was statistical significance (*p* < 0.05) in this issue for men, who believed in the positive impact of 3D food printing on the environment more than women.

Lupton and Turner [15] and Lupton [32] note that 3D food printing could have beneficial effects on the environment, for example, through the reuse of food material (e.g., collagen from tendons, bones, and animal skins) and the development of edible packaging. Some research suggests that 3D food printing could be detrimental to sustainability efforts if it leads to an increase in the transport of food used to make 3D matrices. Furthermore, the sustainability of printed food may be affected by the additional energy required to print the food and to process the materials [33,34]. 

This can affect the affordability of printed food and the adoption of new technologies [35]. Nearly half of the respondents believed that 3D food printing would cause job losses. In particular, statistical significance (*p* < 0.05) was recorded for the group of respondents with a basic education, with a secondary education, with a high school diploma, and for respondents aged under 20 years. We can therefore assume a negative attitude, especially for consumers who perceive 3D printing as a threat to their financial income.

**Figure 9:**
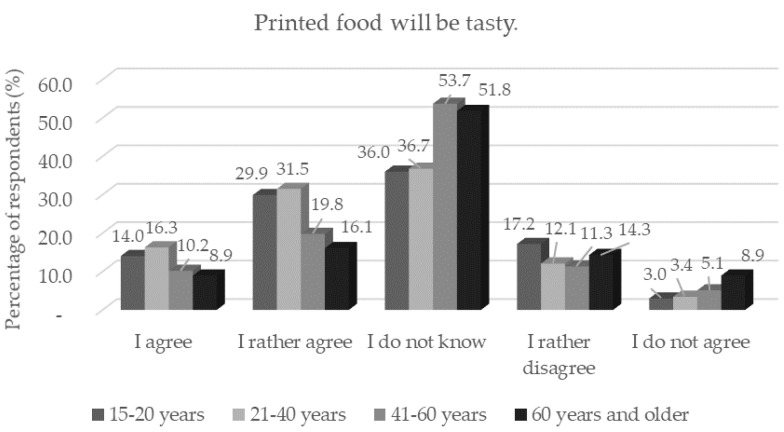
Respondents’ worries and understandings (*n* = 1156) by age category in response to the statement: “Printed food will be tasty.”.

More than half of the respondents confirmed that 3D-printed food would be visually appealing and more than two thirds of the respondents that they would taste 3D-printed food. There was statistical significance (*p* < 0.05) for respondents aged 60 and over who would not taste printed food (Figure 9). This result corresponds to the result of the question whether printed foods will be tasty, where scepticism manifested itself statistically significantly (*p* < 0.05) more in older respondents (41 years and older) (*p* < 0.05) than in younger age categories. The results were consistent with previous studies that younger consumers are better at adopting new technologies [36] and are more innovative than older consumers [37]. A statistically significant (*p* < 0.05) skeptical view of the palatability of printed foods was observed more in women (*n* = 160) than in men (*n* = 50). However, in the study by Caulier et al. [17], it was shown that the repeated consumption of 3D-printed foods can lead to increased consumer acceptance of this technology.

A large number of respondents would taste printed food; conversely, a large number of respondents would not buy printed food (Table 3). Compared with men, women are statistically significantly (*p* < 0.05) more distrustful of these products, which can be expected with regard to the previous questions. The same trend is observed in the group of married respondents. The reluctance to buy printed foods was related to the view that dishes prepared at home are healthier [38], with which the respondents agreed. Statistical significance (*p* < 0.05) was also observed here in women. Several studies show that eating habits are influenced by respondents’ traditions and upbringing [39,40], primarily in women. Women may be affected by traditional food preparation, which they remember from childhood and trust more food prepared at home regardless of their nutritional value [41,42]. In the study [43], the authors report that respondents preferred foods labeled as 3D-printed compared with foods labeled as conventional (off-the-shelf).

**Figure 10:**
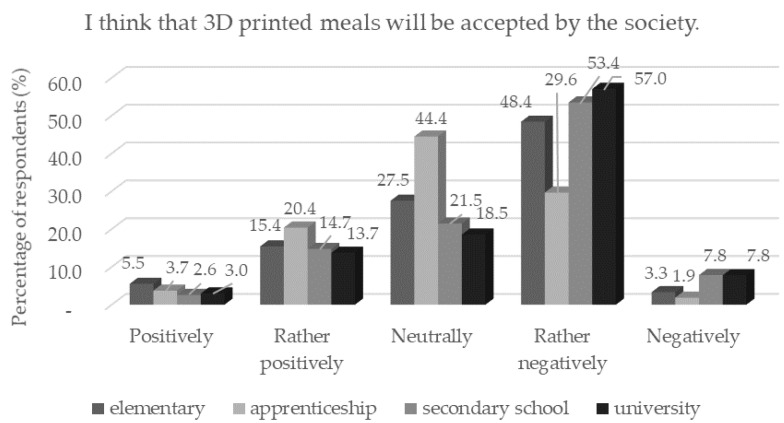
Respondents’ worries and understandings (*n* = 1156) by education in response to the statement: “I think that 3D printed meals will be accepted by society.”.

The acceptance of certain innovative food technologies depends on a complex perception, including cognitive, political, economic, and social aspects [35]. A small number (Table 3) of respondents confirmed that 3D food printing would be received positively by society. Low confidence in the acceptance of 3D printing by society may be related to the fact that new technologies are perceived as risky due to their technical-/chemical-sounding names, which can evoke negative associations [35]. More respondents with a secondary education than respondents with a higher education (*p* < 0.05) believed in the positive acceptance of this food production technology (Figure 10). The percentage of respondents to each question is shown in Table 3.

### 3.4. Three-Dimensional Food Printing, Application

Three-dimensional printing was invented mainly to create an object with a preferred design [44]. The vast majority of respondents (Table 4) stated that 3D food printing will be used to create objects of complex and attractive shapes and that it could be used in confectionery. The 3D printing of chocolate objects is very popular and chocolate appears to be an ideal material for 3D food printing [45]. Currently, there are a number of 3D printers that are designed for printing confectionery and bakery objects, such as Mycusini (Procusini, Germany), Choc Creator V2.0 Plus (Choc Edge Ltd., Exeter, UK), QiaoKe chocolate printer (3DCloud, China), and Fab@Home Model 3 (Creative Machines Lab, New York, NY, USA) [30]. In a study by Chirico Scheele et al. [46], the authors studied the preferences of 3D-printed objects made of marzipan and chocolate. Respondents rated the complex shapes created by 3D printing as unique and aesthetically very attractive. Thanks to the use of 3D printing in the confectionery industry, consumers’ confidence in this technology could increase significantly. More than half (Table 4) of the respondents said that 3D food printing could be used using non-traditional food materials such as insects, algae, etc., which is more than the study by Mantihal [18].

**Figure 11:**
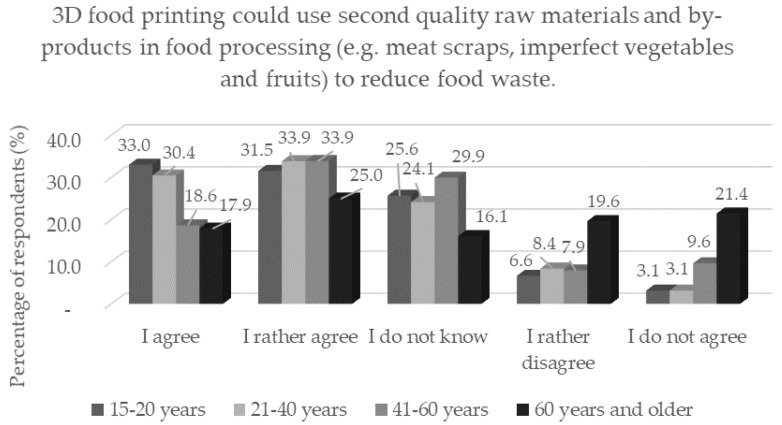
Respondents’ opinions on the application of 3D food printing by age category in response to the statement: “3D food printing could use second quality raw materials and by-products in food processing (e.g., meat scraps, imperfect vegetables and fruits) to reduce food waste.”.

Insect consumption is perceived in society rather as food for developing countries; in Western advanced civilizations it is associated with negative emotions and disgust [47]. Three-dimensional printing can help create a meal from this food material that will arouse the interest of consumers who currently despise it [48]. Based on the results of the questionnaire, we can expect an increase in interest in 3D-printed food from these non-traditional materials, either out of curiosity or conviction. There were statistically significantly (*p* < 0.05) more skeptical respondents aged 60 and over. Older people have been found to be more sensitive to food [49] and thus some disgust can be expected from potential consumption of food printed from insects or with the addition of insects. For the statement of whether 3D food printing could be used in the processing of raw materials of second grades and by-products from the food industry, a similar distribution of respondents (Table 4) was observed as in the previous statement. Older respondents aged 60 years and older were also statistically significantly (*p* < 0.05) more skeptical than the younger age groups (Figure 11). In contrast, men and respondents with single status were statistically significantly (*p* < 0.05) more inclined to this view. Although the use of second-quality raw materials and by-products as food materials may be associated with negative emotions for many consumers, 3D printing can create food that is nutritionally and sensory appealing. In a study by Jagadiswaran et al. [50], the authors explain a new and sustainable approach for the use of grape marc as waste in 3D biscuit printing.

In addition to the use of non-traditional materials and second-quality raw materials, 3D printing could increase the interest in fish meat consumption by creating a bone-free product. Almost half of the respondents (Table 4) believed in this possibility of use, but respondents with a university degree were statistically significantly (*p* < 0.05) more skeptical about this possibility of use than in other categories of education. Fish, especially saltwater fish, are rich in omega-3 fatty acids. Consumption of fish brings a number of health benefits and they become an important protein component in the diet. Currently, a number of research are focusing on the development of a 3D matrix from surimi gel, which appears to be a very good material for 3D printing [51]. Surimi dishes could be a good carrier of fish oil and other biologically active substances and thus contribute to consumers’ health [52].

**Figure 12:**
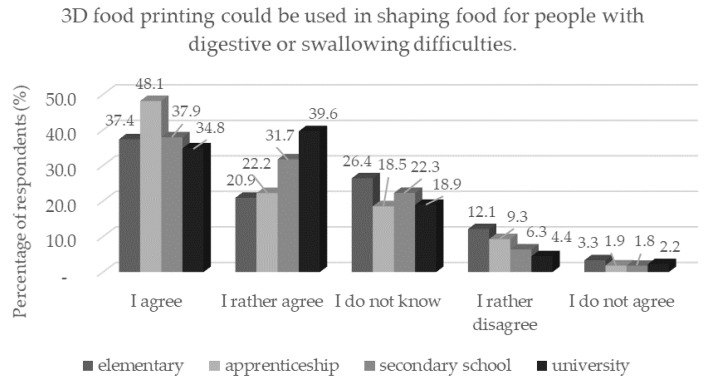
Respondents’ opinions on the application of 3D food printing by education in response to the statement: “3D food printing could be used in shaping food for people with digestive or swallowing difficulties.”.

The majority of respondents in the age category of 60 years and older statistically significantly (*p* < 0.05) did not agree that 3D food printing could be used in the preparation of the required amount of food or food with a precisely defined content of nutrients. However, a large number of respondents were positive about this form of 3D printing. A large number of respondents also agreed with the use of 3D printing in healthcare to create meals for patients on a certain diet and for people with digestive and swallowing problems. Compared with the study of Mantihal [18], an increase in confidence in this area can be observed. However, this comparison could be influenced by the fact that the study of Mantihal [18] was conducted in Australia not in the Czech Republic. In terms of education, respondents with an elementary education did not trust this application statistically significantly (*p* < 0.05) (Figure 12). Patients with swallowing difficulties require a diet containing foods that are soft and safe to swallow. These dishes are usually served in the form of an unsightly porridge. The unpleasant visual aspect of the food can lead to a reduced intake and malnutrition. This problem can be solved by using 3D printing. An example is the study by Pant et al. [53] that deals with the processing of fresh vegetables using 3D printing for application in hospitals, nursing homes, etc. Almost a third of respondents (Table 4) agreed that 3D printing should be used to simplify and speed up food preparation at home. As a result, there was some concern about the new technology and the preference for traditional home-cooked food. On the contrary, almost two thirds of respondents (Table 4) agreed that 3D printing could be used where conditions are difficult for preparing and storing food, such as military camps, space station stays, and in uninhabited areas with extreme climatic conditions.

No statistical significance was observed for these statements (*p* < 0.05). The results indicate that most respondents believe that 3D printing will find its application in places where food preparation is difficult rather than in an environment with a good background. Currently, the US military and NASA (USA) are interested in 3D food printing [54].

Another possibility of using 3D printing is to use it to strengthen social ties through the delivery of food messages, such as a message written in chocolate. Half of the respondents agreed with this use. A study by Wei et al. [55] reports the impact of food messaging (e.g., “Be happy every day”, “You’re the best”, etc.) using a 3D printer. Its results suggest that consumers are pleased with such food messages and perceive them more intensely than text messages. It is therefore clear that 3D food printing can strengthen social ties and communication between people.

The vast majority of respondents (Table 4) believed that 3D printing would become part of food production in the future.

**Figure 13:**
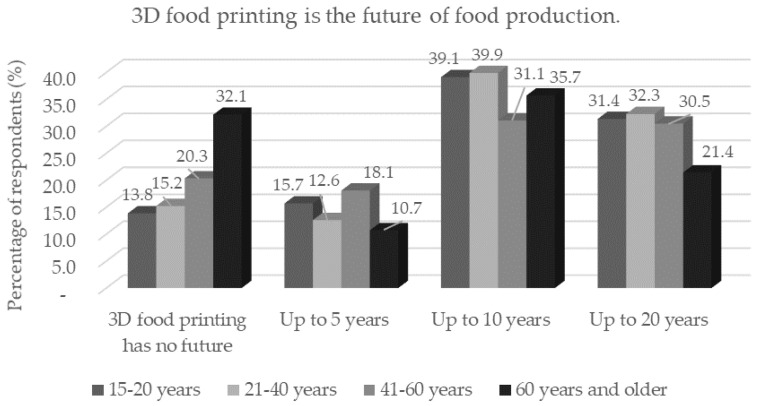
Respondents’ opinions on the application of 3D food printing by age category in response to the statement: “3D food printing is the future of food production.”.

According to the results, it is clear that most respondents believe that 3D printing will be used in the food industry within 10 or 20 years. Respondents over 60 years of age did not believe (*p* < 0.05) in the useful use of 3D food printing (Figure 13); the finding is in accordance with previous replies on the questions. Single respondents trust 3D printing statistically significantly (*p* < 0.05) more than married ones. The results of the study by Jayaprakash et al. [56] suggest that the introduction of 3D food printing is realistic; nevertheless, its success depends on location and innovation. In terms of providing functional benefits, 3D systems based on extrusion are the most suitable for consumers. From the technological and economic feasibility, the greatest potential of 3D printing appears to be used for the preparation of personalized nutrition (athletes, patients in hospitals, seniors). The percentage of respondents to each statement is shown in Table 4.

### 3.5. Three-Dimensional Food Printing, Investments

More than two-thirds of respondents (Table 5) stated that they would not buy a 3D food printer as part of their kitchen equipment. The preferable price for the respondents was mainly up to CZK 15,000 (EUR 615, relative to 21 April 2022). It has been observed that women are more reluctant (*p* < 0.05) to invest in a 3D food printer than men. The same attitude was observed in respondents with a university degree (*p* < 0.05). In terms of age, the willingness to invest in a 3D food printer decreases with age. The percentage of respondents to each question is shown in Table 5. In the study [57], the respondents stated that the application of a 3D food printer in the home environment offers advantages such as risk-free cooking and cooking for self-improvement.

## 4. Conclusions

Sufficient awareness of future consumers is needed to gain confidence in new technologies such as 3D food printing. Although awareness of this technology is increasing, there are still a number of respondents who have not yet encountered or heard of 3D food printing. Three-dimensional food printing is representing a certain concern for respondents, especially when it comes to health benefits, the cost of 3D printed food, or job losses due to the introduction of this technology in food operations. Respondents included in the research mostly perceived printed foods as ultra-processed foods. On the contrary, quality raw materials will be added for their preparation. Respondents believed that printed food would be attractive, and a large proportion of respondents would taste printed food. Most respondents gave a positive assessment of the use of 3D printing in healthcare, the confectionery industry, the military, and hard-to-reach areas. A large number of respondents perceived 3D food printing as a way of processing non-traditional food materials. In terms of socio-demographic data, more men than women seem to be interested in 3D food printing and its applications. Women are also more distrustful of this new technology. A similar distrust was observed among respondents with married status. Awareness and confidence in 3D food printing applications is declining with the growing age of respondents. For the successful introduction of 3D food printing in food operations, and therefore for the introduction of this technology as part of kitchen appliances or as part of catering facilities in public spaces, greater consumer awareness is needed, not only through media and consumer questionnaires but also through personal contact with 3D-printed food. The research emphasized the issue of 3D food printing among respondents in the Czech Republic; the findings gained in the research will certainly be good guidance for further research, as well as for the application of 3D food printing in the preparation of different food commodities.

## Figures and Tables

**Table 1:** Respondents’ socio-demographic properties.

SOCIODEMOGRAPHIC DATA		*n* ^1^	%^2^
**SEX**	woman	**746**	**64.5**
	man	**410**	**35.5**
**MARITAL STATUS**	single	**911**	**78.8**
	married	**245**	**21.2**
**AGE GROUP**	15–20 years	**542**	**46.9**
	21–40 years	**381**	**33.0**
	41–60 years	**177**	**15.3**
	60 years and older	**56**	**4.8**
**EDUCATION**	elementary	**91**	**7.9**
	apprenticeship	**54**	**4.7**
	secondary school	**741**	**64.1**
	university	**270**	**23.4**
**STATUS**	student	**654**	**56.6**
	civil servant	**142**	**12.3**
	private sector employee	**234**	**20.2**
	self-employed	**60**	**5.2**
	retired/disabled person	**46**	**4.0**
	unemployed	**20**	**1.7**
**GROSS INCOME GROUP**	minimum income up to CZK 15,000	**653**	**56.5**
	average income up to CZK 35,000	**369**	**31.9**
	above-average income over CZK 35,000	**134**	**11.6**
**RESIDENCE**	countryside	**432**	**37.4**
	city/town	**724**	**62.6**
**SPECIAL DIET**	none	**983**	**85.0**
	food allergy/intolerance	**70**	**6.1**
	vegetarianism	**59**	**5.1**
	vegan	**13**	**1.1**
	other	**31**	**2.7**

^1^ *n*—number of respondents; ^2^ %—percentage of respondents.

**Table 2:** Three-Dimensional Common Printing Awareness, 3D Food Printing Awareness. Distribution of respondents (%).

	Distribution of Respondents (%)			
	Yes	No		
Have you ever heard of 3D printing?	90.6	9.4		
Are you interested in 3D printing, e.g., by searching for references in the scientific or professional literature?	19.5	80.5		
Have you ever heard of 3D food printing?	38.8	61.2		
Have you ever encountered 3D-printed food stuff?	Yes (internet, television, magazine, etc.)	Yes (shops, restaurants, exhibitions, conferences, etc.)	I do not know	No
	10.3	1.5	21.5	66.7

**Table 3:** Three-Dimensional Food Printing, Worries and Understanding. Distribution of respondents (%).

	Distribution of Respondents (%)				
	I Agree/Yes	I Rather Agree/Rather Yes	I Do Not Know	I Rather Disagree/Rather Not	I Do Not Agree/Not
Quality ingredients will be used to prepare the meals.	29.8	22.6	29.0	14.4	4.2
Three-dimensional food will be healthy.	12.5	13.1	43.4	22.4	8.6
I think that 3D-printed dishes are industrially ultra-processed foods.	25.7	30.3	33.2	7.7	3.1
Food preparation using a 3D printer is not harmful to health and the prepared food is safe to eat.	15.0	25.5	38.6	16.3	4.6
There is a risk of microbial contamination of printed food when using a 3D printer.	7.7	19.9	44.3	21.6	6.5
There is a risk of chemical contamination of printed food when using a 3D printer.	7.2	26.0	38.0	21.6	7.2
Additives will be used in the preparation of 3D printed meals in larger quantities than with food produced by traditional technologies.	11.1	21.2	28.1	18.3	21.3
I think 3D printing can increase the shelf life of food.	16.1	36.0	29.7	13.8	4.4
I think that 3D printing will make food cheaper by reducing production and supply costs.	9.3	23.3	25.1	31.7	10.6
I think that 3D-printed foods are environmentally friendly.	8.5	18.1	43.5	22.3	7.6
I think that 3D printing will have fewer jobs in the food industry.	19.1	33.6	21.6	19.7	6.0
I think 3D-printed dishes will be visually appealing.	28.9	36.0	16.5	15.1	3.5
Printed food will be tasty.	13.9	28.3	39.7	14.4	3.7
I would taste 3D-printed food.	43.1	34.2	7.1	9.4	6.2
I would buy 3D-printed food.	17.0	20.8	16.7	29.0	16.5
	Positively	Rather Positively	Neutrally	Rather Negatively	Negatively
I think that home-cooked food is healthier.	2.9	14.8	22.3	52.8	7.2

**Table 4:** Three-Dimensional Food Printing, Application. Distribution of respondents (%).

	Distribution of Respondents (%)				
	I Agree/Yes	I Rather Agree/Rather Yes	I Do Not Know	I Rather Disagree/Rather Not	I Do Not Agree/Not
Three-dimensional food printing could be used to create complex and attractive shapes.	46.8	32.9	13.9	4.6	1.8
Three-dimensional food printing could have an application in confectionery.	58.5	29.4	8.5	2.2	1.4
Three-dimensional food printing could be used in the use of non-traditional food materials, such as proteins from insects, algae, etc.	35.2	31.2	24.1	5.4	4.1
Three-dimensional food printing could use second quality raw materials and by-products in food processing (e.g., meat scraps, imperfect vegetables and fruits) to reduce food waste.	29.2	32.4	25.3	8.1	5.0
Three-dimensional food printing could be used in the preparation of fish dishes by creating a completely boneless dish and thus increase interest in eating fish meat.	23.4	27.5	27.4	15.9	5.8
Three-dimensional food printing could be used in the preparation of the required amount of food or food with a precisely defined content of nutrients (proteins, amino acids, fats, etc.).	38.9	30.4	21.5	6.9	2.3
Three-dimensional food printing could be used in shaping food for people with digestive or swallowing difficulties.	37.6	32.3	21.6	6.5	2.0
Three-dimensional food printing could be used to simplify and speed up food preparation at home.	14.9	19.2	27.4	26.7	11.8
Three-dimensional food printing could be used in conditions difficult to prepare and store food, such as military camps, staying in a space station, during interplanetary flights, and settling other planets.	49.9	29.5	12.4	5.1	3.1
Three-dimensional food printing could be used to strengthen links in social communication through the online delivery of food messages with a wide range of foods, such as a chocolate object engraved with “All the best”.	21.2	28.2	26.5	16.6	7.5
	Three-dimensional food printing has no future	Up to 5 years	Up to 10 years	Up to 20 years	
Three-dimensional food printing is the future of food production.	16.2	14.7	38.0	31.1	

**Table 5:** Three-Dimensional Food Printing, Investments. Distribution of respondents (%).

	Distribution of Respondents (%)				
	Yes	Rather Yes	I Do Not Know	Rather Not	Not
Would you buy a 3D food printer as part of your kitchen equipment?	6.1	8.0	17.0	29.1	39.8
	I would not invest in a 3D printer.	Max CZK 7000 (Max EUR 285)	7000–15,000 CZK (EUR 285–615)	Min 15,000 CZK (Min EUR 615)	
How much money would you invest in buying a 3D food printer?	59.9	18.2	19.2	2.7	

## Data Availability

The data presented in this study are available within the article.

## Supplementary Materials

The following supporting information can be downloaded at: https://www.mdpi.com/article/10.3390/foods11203154/s1.
