# Supplementary material for: Acceptability Analysis of 3D-Printed Food in the Area of the Czech Republic Based on Survey
Source: Foods. 2022 Oct 11;11(20):3154. doi: 10.3390/foods11203154 (PMC9601544; doi:10.3390/foods11203154)
Supplement: Supplementary file 1 [file foods-11-03154-s001.zip › foods-1891077-supplementary.pdf]

Acceptability of 3D Printed Food in the Czech Republic

*Povinné pole

1. Sex *

Označte jen jednu elipsu.

- woman
- man

2. Marital status *

Označte jen jednu elipsu.

- single
- married

3. Age group *

Označte jen jednu elipsu.

- 15-20 years
- 21-40 years
- 41-60 years
- 60 years and older

4. Education *

Označte jen jednu elipsu.

- elementary
- apprenticeship
- secondary school
- university

5. Status: *

Označte jen jednu elipsu.

- student
- civil servant
- private sector employee
- self employed
- retired / disabled person
- unemployed

6. Gross income group *

Označte jen jednu elipsu.

- minimum income up to CZK 15,000
- average income up to CZK 35,000
- above-average income over CZK 35,000

7. Residence *

Označte jen jednu elipsu.

- countryside
- city/town

8. Special diet *

Označte jen jednu elipsu.

- none
- food allergy/intolerance
- vegetarianism
- vegan
- other

3D Common Printing Awareness

9. Have you ever heard of 3D printing? *

Označte jen jednu elipsu.

- Yes
- No

10. Are you interested in 3D printing? E.g. by searching for references, scientific or professional literature. *

Označte jen jednu elipsu.

- Yes
- No

3D Food Printing Awareness

11. Have you ever heard of 3D food printing? *

Označte jen jednu elipsu.

- Yes
- No

12. Have you ever encountered a 3D printed food stuff? *

Označte jen jednu elipsu.

- Yes (internet, television, magazine, etc.)
- Yes (shops, restaurants, exhibitions, conferences, etc.)
- I do not know
- No

3D Food Printing – Worries and Understanding

13. Quality ingredients will be used to prepare the meals. *

Označte jen jednu elipsu.

- I agree/ Yes
- I rather agree/ Rather yes
- I do not know
- I rather disagree/ Rather not
- I do not agree/ Not

14. 3D food will be healthy *

Označte jen jednu elipsu.

- I agree/ Yes
- I rather agree/ Rather yes
- I do not know
- I rather disagree/ Rather not
- I do not agree/ Not

15. I think that 3D printed dishes are industrially ultra-processed foods. *

Označte jen jednu elipsu.

- I agree/ Yes
- I rather agree/ Rather yes
- I do not know
- I rather disagree/ Rather not
- I do not agree/ Not

16. Food preparation using a 3D printer is not harmful to health and the prepared food is safe to eat. *

Označte jen jednu elipsu.

- I agree/ Yes
- I rather agree/ Rather yes
- I do not know
- I rather disagree/ Rather not
- I do not agree/ Not

17. There is a risk of microbial contamination of printed food when using a 3D printer. *

Označte jen jednu elipsu.

- I agree/ Yes
- I rather agree/ Rather yes
- I do not know
- I rather disagree/ Rather not
- I do not agree/ Not

18. There is a risk of chemical contamination of printed food when using a 3D printer. . *

Označte jen jednu elipsu.

- I agree/ Yes
- I rather agree/ Rather yes
- I do not know
- I rather disagree/ Rather not
- I do not agree/ Not

19. Additives will be used in the preparation of 3D printed meals in larger quantities than with food produced by traditional technologies. *

Označte jen jednu elipsu.

- I agree/ Yes
- I rather agree/ Rather yes
- I do not know
- I rather disagree/ Rather not
- I do not agree/ Not

20. I think 3D printing can increase the shelf life of food *

Označte jen jednu elipsu.

- I agree/ Yes
- I rather agree/ Rather yes
- I do not know
- I rather disagree/ Rather not
- I do not agree/ Not

21. I think that 3D printing will make food cheaper by reducing production and supply costs. *

Označte jen jednu elipsu.

- I agree/ Yes
- I rather agree/ Rather yes
- I do not know
- I rather disagree/ Rather not
- I do not agree/ Not

22. I think that 3D printed foods are environmentally friendly. *

Označte jen jednu elipsu.

- I agree/ Yes
- I rather agree/ Rather yes
- I do not know
- I rather disagree/ Rather not
- I do not agree/ Not

23. I think that 3D printing will have fewer jobs in the food industry. *

Označte jen jednu elipsu.

- I agree/ Yes
- I rather agree/ Rather yes
- I do not know
- I rather disagree/ Rather not
- I do not agree/ Not

24. I think 3D printed dishes will be visually appealing. *

Označte jen jednu elipsu.

- I agree/ Yes
- I rather agree/ Rather yes
- I do not know
- I rather disagree/ Rather not
- I do not agree/ Not

25. Printed food will be tasty. *

Označte jen jednu elipsu.

- I agree/ Yes
- I rather agree/ Rather yes
- I do not know
- I rather disagree/ Rather not
- I do not agree/ Not

26. I would taste 3D printed food. *

Označte jen jednu elipsu.

- I agree/ Yes
- I rather agree/ Rather yes
- I do not know
- I rather disagree/ Rather not
- I do not agree/ Not

27. I would buy a 3D printed food. *

Označte jen jednu elipsu.

- I agree/ Yes
- I rather agree/ Rather yes
- I do not know
- I rather disagree/ Rather not
- I do not agree/ Not

28. I think that home-cooked food is healthier. *

Označte jen jednu elipsu.

- Positively
- Rather positively
- Neutrally
- Rather negatively
- Negatively

3D Food Printing – Application

29. 3D food printing could be used to create complex and attractive shapes. *

Označte jen jednu elipsu.

- I agree/ Yes
- I rather agree/ Rather yes
- I do not know
- I rather disagree/ Rather not
- I do not agree/ Not

30. 3D food printing could have an application in confectionery. *

Označte jen jednu elipsu.

- I agree/ Yes
- I rather agree/ Rather yes
- I do not know
- I rather disagree/ Rather not
- I do not agree/ Not

31. 3D food printing could be used in the use of non-traditional food materials, such as proteins from insects, algae, etc. *

Označte jen jednu elipsu.

- I agree/ Yes
- I rather agree/ Rather yes
- I do not know
- I rather disagree/ Rather not
- I do not agree/ Not

32. 3D food printing could use second quality raw materials and by-products in food processing (e.g. meat scraps, imperfect vegetables and fruits) to reduce food waste. *

Označte jen jednu elipsu.

- I agree/ Yes
- I rather agree/ Rather yes
- I do not know
- I rather disagree/ Rather not
- I do not agree/ Not

33. 3D food printing could be used in the preparation of fish dishes by creating a completely boneless dish and thus increase interest in eating fish meat. *

Označte jen jednu elipsu.

- I agree/ Yes
- I rather agree/ Rather yes
- I do not know
- I rather disagree/ Rather not
- I do not agree/ Not

34. 3D food printing could be used in the preparation of the required amount of food or food with a precisely defined content of nutrients (proteins, amino acids, fats, etc.). *

Označte jen jednu elipsu.

- I agree/ Yes
- I rather agree/ Rather yes
- I do not know
- I rather disagree/ Rather not
- I do not agree/ Not

35. 3D food printing could be used in shaping food for people with digestive or swallowing difficulties. *

Označte jen jednu elipsu.

- I agree/ Yes
- I rather agree/ Rather yes
- I do not know
- I rather disagree/ Rather not
- I do not agree/ Not

36. 3D food printing could be used to simplify and speed up food preparation at home. *

Označte jen jednu elipsu.

- I agree/ Yes
- I rather agree/ Rather yes
- I do not know
- I rather disagree/ Rather not
- I do not agree/ Not

37. 3D food printing could be used in conditions difficult to prepare and store food, such as military camps, staying in a space station, during interplanetary flights, and settling other planets. *

Označte jen jednu elipsu.

- I agree/ Yes
- I rather agree/ Rather yes
- I do not know
- I rather disagree/ Rather not
- I do not agree/ Not

38. 3D food printing could be used to strengthen links in social communication through the online delivery of food messages with a wide range of foods, such as a chocolate object engraved with "All the best". *

Označte jen jednu elipsu.

- I agree/ Yes
- I rather agree/ Rather yes
- I do not know
- I rather disagree/ Rather not
- I do not agree/ Not

39. 3D food printing is the future of food production. *

Označte jen jednu elipsu.

- 3D food print has no future
- Up to 5 years
- Up to 10 years
- Up to 20 years

3D Food Printing – Investments

40. Would you buy a 3D food printer as part of your kitchen equipment? *

Označte jen jednu elipsu.

- Yes
- Rather yes
- I do not know
- Rather not
- Not

41. How much money would you invest in buying a 3D food printer? *

Označte jen jednu elipsu.

- I would not invest in a 3D printer.
- Max CZK 7,000
- 7 000 - 15 000 CZK
- Min 15 000 CZK

Obsah není vytvořen ani schválen Googlem.

Google Formuláře
